# 

**Published:** 1911-11-18

**Authors:** 


### Buildings Contemplated and in Progress.

Sheffield.—Improvements have been carried out at the Sheffield Royal Hospital, consisting of operating-theatres, ophthalmic wards, further administrative offices, and a three-story ward block. The complete hospital has accommodation for 220 patients. Messrs. C. and C. M. Hadfield are the architects.

### GAS FIRES AND RESIDENT STAFFS.

While it is true that some form of central heating is usually fitted nowadays for ward purposes in our voluntary hospitals, the complexity of administration demands that individual fires in the rooms of the various staffs should be readily lit and cleanable. For this purpose gas fires suggest themselves, and any hospital officer who consults the catalogue of, say, the Gas-Light and Coke Company will see designs and applied science of kinds designed to meet every kind of application. The series of small separate coal fires that used to obtain is expensive and administratively difficult. In the quarters of the resident medical officers and in those allotted to the domestic staff gas fires are worth consideration by institutional managers.

### Florence Nightingale Memorial,

### The Blessing of Queen Alexandra's Gift.

We have again the pleasure to record another week of good progress with this memorial. The generous intervention of Queen Alexandra has had a great influence for good, and her example has been followed this week by an increasing number of people and institutions, whose names we publish below. We hope many others may be moved to send in contributions without delay, so that the amount required may be speedily forthcoming. In addition to the names published, a very large number of quite small sums down to 6d. have been sent in. They include one shilling from an old member of the Naval Brigade, who declares that he gladly gives up three ounces of " 'bacca " in testimony of his gratitude to the Lady with the Lamp.

Last week we gave the names of a number of hospitals where the committees and permanent officials are combining to raise a definite sum, and we are glad to publish this week the first of what we hope may be a series of similar collections, from smaller hospitals and institutions. We have to thank Miss Atkey, the matron of the County Hospital, Newport, Mon., for her spontaneous and generous action.

The following contributions are for a statue unless otherwise indicated:—

Lord and Lady Pirrie, £26 5s.; Max Michaelis, Esq., £10 10s.; Sir William Plender, £10 10s.; nurses and residents of County Hospital, Newport, Mon. (per matron), £1 12s. 6d.; Mrs. Hamilton Evans, £1; Miss G. Leigh, 5s.; Received by Mr. G. Q. Roberts, the Hon Sec.: the Worshipful Company of Fishmongers, £105; John Rutherford, Esq., J.P., M.P., £52 10s.; "A Friend of London Hospital Nurses," £50; J. G. Wainwright, Esq., J.P., £25; Joseph H. Choate, Esq., £20; Mrs. Douglas Henty, £20; further from the nursing staff, London Hospital, £12; Mrs. Lloyd Verney, £12; Rt. Hon. the Earl of Listowel, K.P., P.C., £10; Major-Gen. G. A. Crasker, £10; J. S. Neville, Esq., £10; F. W. Burton, Esq., J.P., £5 5s.; D. A. Bevan, Esq., £5 5s.; Messrs. Marnham & Co., £5 5s.; "D.," £5; Mrs. Sword, £5; E. Pinckney, Esq., £5; Mrs. Mary Fitzsimmons, £5; Gen. Sir W. H. Seymour, K.C.B., £5; Miss M. Mitchell, £5; Sir Charles Hartley, K.C.M.G., £5; Rowland Plumbe, Esq., £5; L. Elsdale Molson, Esq., £5; John Abbott, Esq., £5; H. G. Hart, Esq., £5; Theodore Pim, Esq., £5.

Further contributions may be sent to the Honorary Secretary, Florence Nightingale Memorial, St. Thomas's Hospital, Albert Embankment, London, S.E., or to the Editor of The Hospital, 29 Southampton Street, Strand, London, W.C.

### Appointments.

Miss Mary Clarke, M.B., B.S., Birmingham, has been appointed resident medical officer at the Birmingham Children's Hospital, and Miss Hazel Cuthbert, M.B., B.S., takes her place as resident surgical officer.

Mr. Robert W. Macpherson, M.B., B.S., D.P.H., Aberdeen, has been appointed assistant medical officer of health at Chester.

Mr. Hugo Hall has been appointed junior house surgeon at the Coventry and Warwickshire Hospital, and with his appointment the resident staff is complete.

Dr. E. L. Sturdee, formerly casualty surgeon and house physician at the Royal Devon and Exeter, has been appointed medical officer to the Exmouth Dispensary.

The Election Committee of the Liverpool Royal Infirmary, at their meeting on November 8, elected Dr. Frederick P. Wilson, M.S., M.D. (Liverpool), as honorary surgeon to the Department for Specific Diseases.

Dr. Harries has been appointed house surgeon to the special departments at Cardiff Infirmary, and Dr. Rocyn Jones and Dr. Armstrong are to be house surgeons for a further six months. Three honorary assistant anæsthetists are to be appointed.

R. W. Starkie has been appointed clinical assistant to St. Paul's Hospital for Skin Diseases, Red Lion Square.

### The Week's Novelties.

### APONAL.

(Messrs. Zimmer & Co., Frankfort-on-Main. London Agents: Widenmann, Broicher & Co., 33 Lime Street, London, E.C.)

In the *Medizinische Klinik*, some months ago, Huber drew attention to the value of amylene carbamate as a soporific, and pointed out that it was a safe and easily administered drug. Messrs. Zimmer, the well-known Frankfort firm of pharmaceutical chemists, have now put this drug on the market under the name of Aponal. It is obtainable in the form of powder or tablets, both forms being convenient for administration. We have tested samples of the preparation, which appears to us to be an efficient and pleasant hypnotic. The dose is from fifteen to thirty grains; larger quantities should not be prescribed as they appear to cause intoxication. No unpleasant after-effects are produced. The drug is certainly worth a trial by those who prefer a safer soporific than veronal and its allies.

### MALVERN WATERS.

(W. and J. Burrow, The Springs, Malvern.)

We have received samples of the waters bottled by this firm. They include syphons of soda, lithia, and potash water; lemonade made from the natural water, lithia water, table water, and dry ginger-ale. All the samples are free from contamination, and the water is in every case pleasant and agreeable to the taste with the fine flavour and sparkling qualities of the Malvern waters generally. It is the fashion to use continental and foreign table waters, but those who are not prejudiced in their favour should at least try the brands that such well-known firms as this put on the market—brands of thoroughly pure, excellently bottled, and highly palatable English waters which deserve a place on every Englishman's table. The Malvern waters need only to be known to become popular, owing to their limpid purity, their pleasant sparkle, and their natural properties which make them one of the most wholesome table waters with which we are acquainted.

### THE NEW COÇOA.

Eugen Sandow, Ltd., New Kent Road, S.E.

The Sandow Company has turned its attention from physical culture by means of graduated exercise to include physical culture by means of a new cocoa. This is, from the analyst's account, a pure cocoa, one of the insisted on features of which is that it is extremely finely ground, and therefore very soluble. The analysis shows 25 per cent. of albuminoids, 41 per cent. of cocoa extractives, and 13 per cent. of cocoa fat. The public, however, is likely to be influenced by its taste and aroma, and in these it will not be disappointed.

